# 1-Benzyl-3-[3-(naphthalen-2-yl­oxy)prop­yl]imidazolium hexa­fluoro­phosphate

**DOI:** 10.1107/S1600536811026663

**Published:** 2011-07-16

**Authors:** Kun Huang, Bin-Xin Du, Chang-Lu Liu

**Affiliations:** a Department of Chemistry and Chemical Engineering, Sichuan University of Arts and Science, Dazhou 635000, People’s Republic of China; b Sichuan University of Arts and Science, Dazhou 635000, People’s Republic of China

## Abstract

In the title salt, C_23_H_23_N_2_O^+^·PF_6_^−^, the PF_6_^−^ anion is highly disordered (occupancy ratios of 0.35:0.35:0.3, 0.7:0.15:0.15, 0.7:0.3 and 0.35:0.35:0.15:0.15) with the four F atoms in the equatorial plane rotating about the axial F—P—F bond. The mean plane of the imidazole ring makes dihedral angles of 82.44 (17) and 14.39 (16)°, respectively, with the mean planes of the benzene ring and the naphthalene ring system. The crystal structure is stabilized by C—H⋯F hydrogen bonds. In addition, π–π [centroid–centroid distances = 3.7271 (19)–3.8895 (17) Å] and C—H⋯π inter­actions are observed.

## Related literature

For the first free carbenes isolated, see: Arduengo *et al.* (1991 ▶). For applications of *N*-heterocyclic carbene ligands in transmetalation, see: Lin *et al.* (2009 ▶); Wang, Song *et al.* (2005 ▶); Wang, Xu *et al.* (2005 ▶). For the synthesis of the title compound, see: Corma *et al.* (2004 ▶). For related structures, see: Wang, Song *et al.* (2005 ▶). For standard bond lengths, see: Allen *et al.* (1987 ▶).
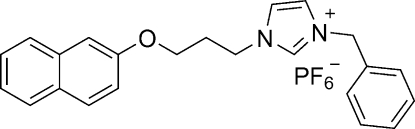


## Experimental

### 

#### Crystal data

C_23_H_23_N_2_O^+^·PF_6_^−^
*M* *_r_* = 488.40
Monoclinic, 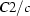

*a* = 28.3309 (5) Å
*b* = 10.2447 (2) Å
*c* = 20.0969 (4) Å
β = 130.296 (1)°
*V* = 4448.87 (15) Å^3^
*Z* = 8
Mo *K*α radiation
μ = 0.19 mm^−1^
*T* = 296 K
0.20 × 0.20 × 0.15 mm

#### Data collection

Bruker SMART CCD area-detector diffractometer
Absorption correction: multi-scan (*SADABS*; Bruker, 2001 ▶) *T*_min_ = 0.963, *T*_max_ = 0.972
19608 measured reflections
5095 independent reflections
3915 reflections with *I* > 2σ(*I*)
*R*_int_ = 0.031

#### Refinement

*R*[*F* ^2^ > 2σ(*F* ^2^)] = 0.057
*wR*(*F* ^2^) = 0.145
*S* = 1.04
5095 reflections
371 parameters
210 restraints
H-atom parameters constrained
Δρ_max_ = 0.35 e Å^−3^
Δρ_min_ = −0.39 e Å^−3^

### 

Data collection: *SMART* (Bruker, 2007 ▶); cell refinement: *SAINT* (Bruker, 2007 ▶); data reduction: *SAINT*; program(s) used to solve structure: *SHELXS97* (Sheldrick, 2008 ▶); program(s) used to refine structure: *SHELXL97* (Sheldrick, 2008 ▶); molecular graphics: *SHELXTL* (Sheldrick, 2008 ▶); software used to prepare material for publication: *SHELXTL*.

## Supplementary Material

Crystal structure: contains datablock(s) global, I. DOI: 10.1107/S1600536811026663/lr2017sup1.cif

Structure factors: contains datablock(s) I. DOI: 10.1107/S1600536811026663/lr2017Isup2.hkl

Supplementary material file. DOI: 10.1107/S1600536811026663/lr2017Isup3.cml

Additional supplementary materials:  crystallographic information; 3D view; checkCIF report

## Figures and Tables

**Table 1:** Hydrogen-bond geometry (Å, °) *Cg*2 is the centroid of the C1–C4/C9/C10 ring.

*D*—H⋯*A*	*D*—H	H⋯*A*	*D*⋯*A*	*D*—H⋯*A*
C16—H16⋯F2^i^	0.93	2.41	3.304 (3)	160
C19—H19⋯F2^ii^	0.93	2.49	3.393 (3)	162
C14—H14⋯F4*A*	0.93	2.48	3.406 (6)	171
C4—H4⋯F5*A*^iii^	0.93	2.55	3.315 (11)	140
C13—H13*B*⋯*Cg*2^iv^	0.97	2.65	3.560 (3)	157

Symmetry codes: (i) 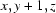; (ii) 
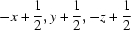
; (iii) 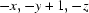; (iv) 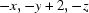.

**Fig. 1.**
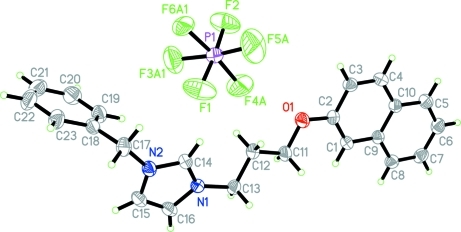
A view of the molecular structure of the title compound with atom numbering. The displacement ellipsoids are drawn at the 30% probability level. Only part of the disordered PF6- atoms are shown.

**Fig. 2.**
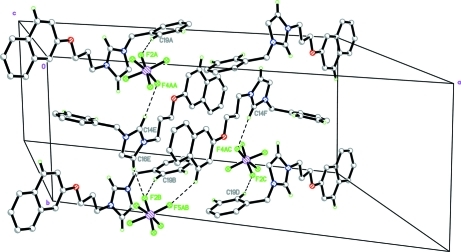
Part of crystal packing of the title compound, showing the cations and anions linked via C—H···F interactions (dashed lines). H atoms not involved in these interactions have been omitted for clarity.

## supplementary crystallographic information

### Comment

The discovery of the first free N-heterocyclic carbene (NHC) was disclosed by Arduengo and coworks (Arduengo *et al.*, 1991). 1,3-disubstituted imidazolium salts are widely used as precursors for the synthesize of transition metal NHC's (Lin *et al.*, 2009; Wang, Song *et al.*, 2005; Wang, Xu *et al.*, 2005). Herein we report on the crystal structure of the title compound, a new NHC precursor.

The molecular structure of the title compound is shown in Fig. 1. Bond lengths (Allen *et al.*, 1987) and angles in the cation are normal. The mean plane of the imidazole ring makes dihedral angles with the mean planes of the benzene and naphthalene rings of 82.44 (17)° and 14.39 (16)°, respectively. The PF_6_^-^ is disordered with as many as three sites found for some of the four F atoms (F3—F6) in the equatorial plane.

In the crystal there are weak π–π interactions involving the imidazole and naphthalene rings with centroid-centroid distances, *Cg*1···*Cg*3^i^, *Cg*2···*Cg*3^ii^ and *Cg*2···*Cg*2^ii^ of 3.731 (2), 3.7271 (19) and 3.8895 (17) Å, respectively [symmetry codes: (i) -*x*, 2 - *y*, -*z*; (ii) -*x*, *y*, -1/2 -*z. Cg*1 centroid of the imidazole ring (N1,N2,C14—C16); *Cg*2 centroid of ring (C1—C4,C9,C10); *Cg*3 centroid of ring (C5—C10)]. In addition, C—H···F hydrogen bonds and C—H···π interactions are observed (Table 1 and Fig. 2).

### Experimental

The title compound was prepared according to the reported procedures (Corma *et al.*, 2004). Colourless single crystals suitable for X-ray diffraction were obtained by recrystallization from acetonitrile and ethyl ether (v:v = 1:1).

### Refinement

H atoms were placed in calculated orientations and treated as riding atoms: C—H =0.93 and 0.97 Å, for CH and CH_2_ H-atoms, respectively, with *U*_iso_(H) =1.2*U*_eq_(C). The PF_6_ anion is highly disordered and atoms F3—F6 were split(occupancies: 0.7 for F4A,F5A; 0.35 for F3A1,F3A2, F6A1,F6A2,F3B,F5B; 0.15 for F4B1,F4B2,F6B1,F6B2) and refined with distance restraints of P—F = 1.55 (2) Å. Attempts to further split theses atoms were unsuccessful.

### Crystal data

**Table:**

C_23_H_23_N_2_O^+^·PF_6_^−^	*F*(000) = 2016
*M**_r_* = 488.40	*D*_x_ = 1.458 Mg m^−^^3^
Monoclinic, *C*2/*c*	Mo *K*α radiation, λ = 0.71073 Å
Hall symbol: -C 2yc	Cell parameters from 5985 reflections
*a* = 28.3309 (5) Å	θ = 2.7–27.2°
*b* = 10.2447 (2) Å	µ = 0.19 mm^−^^1^
*c* = 20.0969 (4) Å	*T* = 296 K
β = 130.296 (1)°	Block, colourless
*V* = 4448.87 (15) Å^3^	0.20 × 0.20 × 0.15 mm
*Z* = 8	

### Data collection

**Table:**

Bruker SMART CCD area-detector diffractometer	5095 independent reflections
Radiation source: fine-focus sealed tube	3915 reflections with *I* > 2σ(*I*)
graphite	*R*_int_ = 0.031
φ and ω scans	θ_max_ = 27.5°, θ_min_ = 1.9°
Absorption correction: multi-scan (*SADABS*; Bruker, 2001)	*h* = −36→36
*T*_min_ = 0.963, *T*_max_ = 0.972	*k* = −13→13
19608 measured reflections	*l* = −26→26

### Refinement

**Table:**

Refinement on *F*^2^	Secondary atom site location: difference Fourier map
Least-squares matrix: full	Hydrogen site location: inferred from neighbouring sites
*R*[*F*^2^ > 2σ(*F*^2^)] = 0.057	H-atom parameters constrained
*wR*(*F*^2^) = 0.145	*w* = 1/[σ^2^(*F*_o_^2^) + (0.0515*P*)^2^ + 4.9635*P*] where *P* = (*F*_o_^2^ + 2*F*_c_^2^)/3
*S* = 1.04	(Δ/σ)_max_ = 0.003
5095 reflections	Δρ_max_ = 0.35 e Å^−^^3^
371 parameters	Δρ_min_ = −0.39 e Å^−^^3^
210 restraints	Extinction correction: *SHELXL97* (Sheldrick, 2008), Fc^*^=kFc[1+0.001xFc^2^λ^3^/sin(2θ)]^-1/4^
Primary atom site location: structure-invariant direct methods	Extinction coefficient: 0.00093 (14)

### Special details

**Table:**

Geometry. Bond distances, angles *etc*. have been calculated using the rounded fractional coordinates. All su's are estimated from the variances of the (full) variance-covariance matrix. The cell e.s.d.'s are taken into account in the estimation of distances, angles and torsion angles
Refinement. Refinement of *F*^2^ against ALL reflections. The weighted *R*-factor *wR* and goodness of fit *S* are based on *F*^2^, conventional *R*-factors *R* are based on *F*, with *F* set to zero for negative *F*^2^. The threshold expression of *F*^2^ > σ(*F*^2^) is used only for calculating *R*-factors(gt) *etc*. and is not relevant to the choice of reflections for refinement. *R*-factors based on *F*^2^ are statistically about twice as large as those based on *F*, and *R*- factors based on ALL data will be even larger.

### Fractional atomic coordinates and isotropic or equivalent isotropic displacement parameters (Å^2^)

**Table:**

	*x*	*y*	*z*	*U*_iso_*/*U*_eq_	Occ. (<1)
O1	0.06916 (7)	0.87976 (16)	−0.00405 (10)	0.0616 (5)	
N1	0.20226 (8)	1.10961 (17)	0.27117 (11)	0.0448 (5)	
N2	0.27187 (8)	1.03723 (18)	0.40219 (11)	0.0488 (6)	
C1	−0.00745 (9)	0.9740 (2)	−0.14818 (13)	0.0479 (6)	
C2	0.02224 (10)	0.8706 (2)	−0.09241 (13)	0.0475 (6)	
C3	0.00510 (10)	0.7413 (2)	−0.12412 (15)	0.0521 (7)	
C4	−0.04215 (10)	0.7189 (2)	−0.21032 (15)	0.0535 (7)	
C5	−0.12511 (11)	0.8038 (3)	−0.35933 (15)	0.0605 (8)	
C6	−0.15535 (11)	0.9062 (3)	−0.41528 (15)	0.0659 (9)	
C7	−0.13672 (11)	1.0334 (3)	−0.38480 (15)	0.0611 (8)	
C8	−0.08860 (10)	1.0564 (2)	−0.29828 (15)	0.0547 (7)	
C9	−0.05671 (9)	0.9525 (2)	−0.23861 (13)	0.0447 (6)	
C10	−0.07497 (9)	0.8233 (2)	−0.27019 (13)	0.0473 (7)	
C11	0.08538 (10)	1.0064 (2)	0.03396 (14)	0.0536 (7)	
C12	0.13234 (10)	0.9878 (2)	0.13195 (14)	0.0539 (7)	
C13	0.14909 (9)	1.1174 (2)	0.17730 (13)	0.0490 (7)	
C14	0.22160 (9)	1.0060 (2)	0.32192 (13)	0.0459 (6)	
C15	0.28478 (12)	1.1662 (2)	0.40219 (16)	0.0619 (8)	
C16	0.24136 (11)	1.2109 (2)	0.32116 (16)	0.0600 (8)	
C17	0.30690 (10)	0.9486 (3)	0.47728 (14)	0.0582 (8)	
C18	0.37109 (10)	0.9213 (2)	0.50999 (14)	0.0505 (7)	
C19	0.38433 (11)	0.9188 (3)	0.45503 (16)	0.0627 (9)	
C20	0.44286 (13)	0.8888 (3)	0.4866 (2)	0.0774 (11)	
C21	0.48900 (14)	0.8610 (3)	0.5734 (2)	0.0828 (10)	
C22	0.47645 (13)	0.8651 (3)	0.6284 (2)	0.0845 (10)	
C23	0.41781 (12)	0.8944 (3)	0.59729 (16)	0.0688 (9)	
H1	0.00460	1.05870	−0.12690	0.0580*	
H3	0.02620	0.67140	−0.08600	0.0620*	
H4	−0.05320	0.63340	−0.23040	0.0640*	
H5	−0.13790	0.71910	−0.38050	0.0730*	
H6	−0.18850	0.89110	−0.47400	0.0790*	
H7	−0.15700	1.10310	−0.42340	0.0730*	
H8	−0.07680	1.14190	−0.27850	0.0660*	
H11A	0.04910	1.05140	0.01760	0.0640*	
H11B	0.10310	1.05770	0.01420	0.0640*	
H12A	0.11520	0.93120	0.15040	0.0650*	
H12B	0.16920	0.94650	0.14780	0.0650*	
H13A	0.15860	1.17850	0.15050	0.0590*	
H13B	0.11370	1.15110	0.16920	0.0590*	
H14	0.20300	0.92410	0.30420	0.0550*	
H15	0.31760	1.21370	0.44970	0.0740*	
H16	0.23820	1.29570	0.30210	0.0720*	
H17A	0.28460	0.86680	0.46090	0.0700*	
H17B	0.30990	0.98660	0.52410	0.0700*	
H19	0.35340	0.93770	0.39610	0.0750*	
H20	0.45130	0.88720	0.44890	0.0930*	
H21	0.52850	0.83950	0.59450	0.1000*	
H22	0.50780	0.84800	0.68750	0.1010*	
H23	0.40970	0.89610	0.63530	0.0830*	
P1	0.19683 (3)	0.60238 (6)	0.31472 (4)	0.0536 (2)	
F1	0.19758 (14)	0.6908 (2)	0.37951 (18)	0.1311 (13)	
F2	0.20206 (13)	0.5170 (2)	0.25553 (14)	0.1257 (12)	
F3A1	0.2634 (5)	0.6501 (17)	0.3723 (11)	0.131 (5)	0.350
F3A2	0.2482 (4)	0.6998 (10)	0.3320 (8)	0.071 (3)	0.350
F3B	0.2173 (9)	0.7164 (9)	0.2875 (11)	0.102 (5)	0.300
F4A	0.1558 (4)	0.7068 (5)	0.2407 (3)	0.148 (2)	0.700
F4B1	0.1325 (7)	0.617 (2)	0.2333 (15)	0.138 (6)	0.150
F4B2	0.1395 (9)	0.665 (3)	0.2819 (19)	0.137 (9)	0.150
F5A	0.1349 (3)	0.5355 (9)	0.2739 (7)	0.221 (4)	0.700
F5B	0.1705 (8)	0.4928 (12)	0.3317 (10)	0.100 (5)	0.300
F6A1	0.2462 (7)	0.5115 (12)	0.3909 (7)	0.097 (4)	0.350
F6A2	0.2118 (8)	0.4825 (11)	0.3762 (7)	0.094 (4)	0.350
F6B1	0.2609 (7)	0.540 (3)	0.3665 (14)	0.104 (7)	0.150
F6B2	0.2639 (11)	0.566 (4)	0.3971 (15)	0.115 (8)	0.150

### Atomic displacement parameters (Å^2^)

**Table:**

	*U*^11^	*U*^22^	*U*^33^	*U*^12^	*U*^13^	*U*^23^
O1	0.0601 (9)	0.0549 (10)	0.0432 (8)	0.0002 (8)	0.0215 (7)	−0.0003 (7)
N1	0.0444 (9)	0.0445 (9)	0.0433 (9)	0.0008 (7)	0.0274 (8)	−0.0019 (7)
N2	0.0472 (9)	0.0547 (11)	0.0414 (9)	−0.0020 (8)	0.0272 (8)	−0.0037 (8)
C1	0.0472 (11)	0.0462 (11)	0.0476 (11)	−0.0061 (9)	0.0294 (10)	−0.0051 (9)
C2	0.0438 (10)	0.0530 (12)	0.0417 (10)	−0.0021 (9)	0.0258 (9)	0.0000 (9)
C3	0.0559 (12)	0.0466 (12)	0.0528 (12)	0.0024 (10)	0.0347 (11)	0.0040 (10)
C4	0.0610 (13)	0.0441 (12)	0.0572 (13)	−0.0051 (10)	0.0390 (12)	−0.0068 (10)
C5	0.0597 (14)	0.0635 (15)	0.0489 (13)	−0.0043 (11)	0.0309 (11)	−0.0132 (11)
C6	0.0550 (13)	0.0847 (19)	0.0429 (12)	0.0063 (13)	0.0249 (11)	−0.0081 (12)
C7	0.0583 (14)	0.0700 (16)	0.0488 (13)	0.0154 (12)	0.0319 (11)	0.0087 (11)
C8	0.0554 (13)	0.0520 (13)	0.0528 (12)	0.0031 (10)	0.0333 (11)	0.0019 (10)
C9	0.0415 (10)	0.0514 (12)	0.0439 (10)	−0.0017 (9)	0.0288 (9)	−0.0017 (9)
C10	0.0460 (11)	0.0530 (12)	0.0453 (11)	−0.0017 (9)	0.0306 (10)	−0.0055 (9)
C11	0.0480 (11)	0.0564 (13)	0.0439 (11)	−0.0020 (10)	0.0241 (10)	−0.0017 (10)
C12	0.0473 (11)	0.0575 (13)	0.0450 (11)	0.0009 (10)	0.0245 (10)	0.0001 (10)
C13	0.0439 (11)	0.0556 (13)	0.0419 (11)	0.0048 (9)	0.0252 (9)	0.0032 (9)
C14	0.0445 (10)	0.0468 (11)	0.0431 (10)	−0.0056 (9)	0.0268 (9)	−0.0046 (9)
C15	0.0651 (15)	0.0518 (13)	0.0556 (14)	−0.0115 (11)	0.0331 (12)	−0.0165 (11)
C16	0.0647 (14)	0.0434 (12)	0.0578 (14)	−0.0062 (11)	0.0333 (12)	−0.0090 (10)
C17	0.0536 (13)	0.0743 (16)	0.0404 (11)	−0.0015 (11)	0.0276 (10)	0.0047 (11)
C18	0.0511 (12)	0.0472 (12)	0.0426 (11)	−0.0058 (9)	0.0255 (10)	−0.0031 (9)
C19	0.0578 (14)	0.0751 (17)	0.0500 (13)	−0.0036 (12)	0.0325 (11)	−0.0086 (12)
C20	0.0726 (17)	0.081 (2)	0.089 (2)	−0.0050 (15)	0.0570 (17)	−0.0115 (16)
C21	0.0587 (16)	0.0665 (18)	0.105 (2)	0.0072 (13)	0.0448 (18)	0.0044 (17)
C22	0.0608 (16)	0.083 (2)	0.0673 (18)	0.0112 (15)	0.0223 (14)	0.0197 (16)
C23	0.0647 (15)	0.0763 (17)	0.0480 (13)	0.0036 (13)	0.0286 (12)	0.0109 (12)
P1	0.0594 (4)	0.0487 (3)	0.0567 (4)	0.0039 (3)	0.0394 (3)	0.0031 (3)
F1	0.214 (3)	0.0915 (15)	0.175 (2)	−0.0149 (16)	0.165 (2)	−0.0274 (15)
F2	0.220 (3)	0.0890 (14)	0.1101 (16)	−0.0037 (15)	0.1257 (18)	−0.0170 (12)
F4A	0.173 (5)	0.086 (3)	0.107 (3)	0.039 (3)	0.055 (3)	0.045 (2)
F5A	0.114 (4)	0.182 (7)	0.290 (9)	−0.075 (4)	0.096 (5)	−0.006 (7)
F3A1	0.084 (5)	0.156 (12)	0.125 (10)	−0.036 (7)	0.055 (6)	−0.045 (8)
F3A2	0.066 (5)	0.075 (6)	0.092 (7)	−0.014 (4)	0.060 (5)	−0.005 (5)
F4B1	0.035 (6)	0.090 (12)	0.118 (14)	0.010 (8)	−0.027 (7)	0.013 (11)
F3B	0.184 (13)	0.053 (4)	0.127 (9)	0.022 (8)	0.127 (9)	0.030 (6)
F4B2	0.069 (10)	0.108 (15)	0.183 (19)	0.034 (10)	0.059 (12)	−0.026 (16)
F6A1	0.113 (9)	0.075 (7)	0.062 (4)	0.030 (7)	0.038 (6)	0.024 (4)
F5B	0.144 (10)	0.087 (7)	0.128 (9)	−0.066 (7)	0.115 (8)	−0.047 (7)
F6A2	0.140 (10)	0.074 (6)	0.075 (6)	0.034 (8)	0.073 (7)	0.030 (5)
F6B1	0.062 (7)	0.120 (14)	0.160 (14)	0.027 (8)	0.085 (9)	0.016 (12)
F6B2	0.106 (13)	0.114 (17)	0.046 (8)	0.003 (12)	0.014 (7)	0.042 (11)

### Geometric parameters (Å, °)

**Table:**

P1—F6A2	1.593 (12)	C7—C8	1.368 (3)
P1—F6B1	1.53 (3)	C8—C9	1.409 (3)
P1—F6B2	1.56 (3)	C9—C10	1.413 (3)
P1—F3A1	1.521 (19)	C11—C12	1.515 (3)
P1—F3A2	1.607 (14)	C12—C13	1.503 (3)
P1—F4B1	1.47 (2)	C15—C16	1.336 (3)
P1—F1	1.575 (3)	C17—C18	1.507 (5)
P1—F2	1.559 (3)	C18—C23	1.379 (3)
P1—F4A	1.572 (5)	C18—C19	1.377 (4)
P1—F5A	1.536 (12)	C19—C20	1.373 (6)
P1—F3B	1.55 (2)	C20—C21	1.372 (4)
P1—F4B2	1.45 (3)	C21—C22	1.363 (6)
P1—F6A1	1.548 (12)	C22—C23	1.378 (6)
P1—F5B	1.504 (19)	C1—H1	0.9300
F1—F4B2	1.57 (3)	C3—H3	0.9300
F3A1—F3A2	0.81 (2)	C4—H4	0.9300
F3A1—F6A1	1.62 (2)	C5—H5	0.9300
F4B1—F4B2	0.99 (4)	C6—H6	0.9300
F6A1—F6A2	0.87 (3)	C7—H7	0.9300
F6B1—F6B2	0.62 (4)	C8—H8	0.9300
O1—C2	1.370 (3)	C11—H11A	0.9700
O1—C11	1.423 (3)	C11—H11B	0.9700
N1—C13	1.469 (3)	C12—H12B	0.9700
N1—C14	1.321 (3)	C12—H12A	0.9700
N1—C16	1.369 (3)	C13—H13A	0.9700
N2—C17	1.466 (3)	C13—H13B	0.9700
N2—C15	1.371 (3)	C14—H14	0.9300
N2—C14	1.327 (3)	C15—H15	0.9300
C1—C2	1.366 (3)	C16—H16	0.9300
C1—C9	1.421 (3)	C17—H17B	0.9700
C2—C3	1.413 (3)	C17—H17A	0.9700
C3—C4	1.359 (3)	C19—H19	0.9300
C4—C10	1.417 (3)	C20—H20	0.9300
C5—C6	1.361 (4)	C21—H21	0.9300
C5—C10	1.406 (3)	C22—H22	0.9300
C6—C7	1.391 (4)	C23—H23	0.9300
			
F6B1—P1—F6B2	23.3 (15)	C1—C9—C10	119.41 (18)
F1—P1—F4B2	62.2 (12)	C8—C9—C10	118.57 (19)
F1—P1—F6A1	90.5 (6)	C1—C9—C8	122.02 (19)
F1—P1—F5B	89.2 (7)	C4—C10—C5	122.8 (2)
F1—P1—F6A2	87.0 (6)	C4—C10—C9	118.48 (19)
F1—P1—F6B1	107.6 (9)	C5—C10—C9	118.7 (2)
F1—P1—F6B2	84.5 (13)	O1—C11—C12	106.88 (16)
F2—P1—F4A	92.1 (3)	C11—C12—C13	110.15 (17)
F2—P1—F5A	89.0 (4)	N1—C13—C12	112.96 (17)
F3A1—P1—F2	94.5 (7)	N1—C14—N2	109.15 (19)
F2—P1—F3A2	89.6 (5)	N2—C15—C16	107.2 (2)
F2—P1—F4B1	82.5 (10)	N1—C16—C15	107.76 (19)
F2—P1—F3B	84.9 (7)	N2—C17—C18	112.7 (2)
F2—P1—F4B2	122.2 (12)	C19—C18—C23	118.8 (3)
F2—P1—F6A1	86.9 (6)	C17—C18—C23	119.1 (3)
F2—P1—F5B	93.5 (7)	C17—C18—C19	122.1 (2)
F2—P1—F6A2	92.9 (6)	C18—C19—C20	120.5 (3)
F2—P1—F6B1	68.2 (9)	C19—C20—C21	120.4 (4)
F2—P1—F6B2	91.4 (13)	C20—C21—C22	119.4 (4)
F4A—P1—F5A	84.7 (5)	C21—C22—C23	120.6 (3)
F3A1—P1—F3A2	29.7 (8)	C18—C23—C22	120.3 (3)
F3A1—P1—F6A1	63.8 (9)	C9—C1—H1	120.00
F3A1—P1—F6A2	94.6 (10)	C2—C1—H1	120.00
F3A2—P1—F6A1	92.7 (8)	C4—C3—H3	120.00
F3A2—P1—F6A2	124.2 (8)	C2—C3—H3	120.00
F1—P1—F2	175.2 (2)	C3—C4—H4	119.00
F1—P1—F4A	89.9 (3)	C10—C4—H4	119.00
F1—P1—F5A	95.6 (4)	C10—C5—H5	119.00
F1—P1—F3A1	80.7 (7)	C6—C5—H5	119.00
F1—P1—F3A2	86.5 (5)	C5—C6—H6	120.00
F1—P1—F4B1	101.9 (10)	C7—C6—H6	120.00
F1—P1—F3B	92.9 (7)	C6—C7—H7	120.00
F6A1—P1—F6A2	32.0 (11)	C8—C7—H7	120.00
F4B1—P1—F6B2	171.8 (17)	C7—C8—H8	120.00
F4B1—P1—F4B2	39.7 (15)	C9—C8—H8	119.00
F4B1—P1—F6B1	150.5 (13)	O1—C11—H11A	110.00
F4B2—P1—F6B2	145.9 (17)	O1—C11—H11B	110.00
F3B—P1—F5B	174.0 (9)	C12—C11—H11A	110.00
F4B2—P1—F6B1	169.0 (15)	C12—C11—H11B	110.00
P1—F1—F4B2	55.0 (12)	H11A—C11—H11B	109.00
P1—F3A1—F6A1	58.9 (9)	H12A—C12—H12B	108.00
F3A2—F3A1—F6A1	138 (2)	C11—C12—H12B	110.00
P1—F3A1—F3A2	81.1 (17)	C11—C12—H12A	110.00
P1—F3A2—F3A1	69.2 (16)	C13—C12—H12A	110.00
P1—F4B1—F4B2	69 (2)	C13—C12—H12B	110.00
P1—F4B2—F1	62.9 (13)	N1—C13—H13B	109.00
F1—F4B2—F4B1	134 (3)	N1—C13—H13A	109.00
P1—F4B2—F4B1	71 (2)	H13A—C13—H13B	108.00
P1—F6A1—F3A1	57.3 (8)	C12—C13—H13A	109.00
P1—F6A1—F6A2	76.8 (12)	C12—C13—H13B	109.00
F3A1—F6A1—F6A2	131.3 (17)	N1—C14—H14	125.00
P1—F6A2—F6A1	71.2 (12)	N2—C14—H14	125.00
P1—F6B1—F6B2	81 (4)	C16—C15—H15	126.00
P1—F6B2—F6B1	76 (3)	N2—C15—H15	126.00
C2—O1—C11	117.66 (17)	C15—C16—H16	126.00
C13—N1—C14	127.16 (18)	N1—C16—H16	126.00
C13—N1—C16	124.91 (18)	N2—C17—H17A	109.00
C14—N1—C16	107.93 (18)	H17A—C17—H17B	108.00
C14—N2—C17	125.6 (2)	C18—C17—H17B	109.00
C15—N2—C17	126.4 (2)	N2—C17—H17B	109.00
C14—N2—C15	107.99 (18)	C18—C17—H17A	109.00
C2—C1—C9	120.23 (19)	C18—C19—H19	120.00
O1—C2—C1	125.23 (19)	C20—C19—H19	120.00
O1—C2—C3	114.29 (18)	C21—C20—H20	120.00
C1—C2—C3	120.48 (19)	C19—C20—H20	120.00
C2—C3—C4	120.1 (2)	C22—C21—H21	120.00
C3—C4—C10	121.29 (19)	C20—C21—H21	120.00
C6—C5—C10	121.4 (3)	C21—C22—H22	120.00
C5—C6—C7	120.1 (2)	C23—C22—H22	120.00
C6—C7—C8	120.2 (2)	C18—C23—H23	120.00
C7—C8—C9	121.0 (2)	C22—C23—H23	120.00
			
C11—O1—C2—C1	5.0 (5)	C10—C5—C6—C7	0.3 (6)
C11—O1—C2—C3	−174.6 (3)	C6—C5—C10—C4	−179.5 (3)
C2—O1—C11—C12	173.4 (3)	C6—C5—C10—C9	1.1 (5)
C14—N1—C13—C12	−24.7 (4)	C5—C6—C7—C8	−1.1 (6)
C16—N1—C13—C12	154.2 (3)	C6—C7—C8—C9	0.5 (5)
C13—N1—C14—N2	178.5 (3)	C7—C8—C9—C1	−178.7 (3)
C16—N1—C14—N2	−0.5 (4)	C7—C8—C9—C10	0.8 (5)
C13—N1—C16—C15	−178.3 (3)	C1—C9—C10—C4	−1.6 (4)
C14—N1—C16—C15	0.8 (4)	C1—C9—C10—C5	177.9 (3)
C15—N2—C14—N1	0.1 (4)	C8—C9—C10—C4	178.9 (3)
C17—N2—C14—N1	179.9 (3)	C8—C9—C10—C5	−1.5 (5)
C14—N2—C15—C16	0.4 (4)	O1—C11—C12—C13	−176.6 (3)
C17—N2—C15—C16	−179.4 (3)	C11—C12—C13—N1	−169.8 (3)
C14—N2—C17—C18	113.9 (3)	N2—C15—C16—N1	−0.7 (4)
C15—N2—C17—C18	−66.4 (4)	N2—C17—C18—C19	−31.7 (3)
C9—C1—C2—O1	−178.4 (3)	N2—C17—C18—C23	150.2 (2)
C9—C1—C2—C3	1.1 (5)	C17—C18—C19—C20	−177.6 (3)
C2—C1—C9—C8	179.9 (3)	C23—C18—C19—C20	0.6 (4)
C2—C1—C9—C10	0.4 (5)	C17—C18—C23—C22	178.0 (3)
O1—C2—C3—C4	178.0 (3)	C19—C18—C23—C22	−0.3 (4)
C1—C2—C3—C4	−1.5 (5)	C18—C19—C20—C21	−0.1 (5)
C2—C3—C4—C10	0.3 (5)	C19—C20—C21—C22	−0.9 (5)
C3—C4—C10—C5	−178.3 (3)	C20—C21—C22—C23	1.2 (5)
C3—C4—C10—C9	1.2 (5)	C21—C22—C23—C18	−0.7 (5)

### Hydrogen-bond geometry (Å, °)

**Table:**

Cg2 is the centroid of the C1–C4/C9/C10 ring.

**Table:**

*D*—H···*A*	*D*—H	H···*A*	*D*···*A*	*D*—H···*A*
C16—H16···F2^i^	0.93	2.41	3.304 (3)	160
C19—H19···F2^ii^	0.93	2.49	3.393 (3)	162
C14—H14···F4A	0.93	2.48	3.406 (6)	171
C4—H4···F5A^iii^	0.93	2.55	3.315 (11)	140
C13—H13B···Cg2^iv^	0.97	2.65	3.560 (3)	157

Symmetry codes: (i) *x*, *y*+1, *z*; (ii) −*x*+1/2, *y*+1/2, −*z*+1/2; (iii) −*x*, −*y*+1, −*z*; (iv) −*x*, −*y*+2, −*z*.
